# Human Amniotic Epithelial Cells and Their Derived Exosomes Protect Against Cisplatin-Induced Acute Kidney Injury Without Compromising Its Antitumor Activity in Mice

**DOI:** 10.3389/fcell.2021.752053

**Published:** 2022-02-03

**Authors:** Xin Kang, Ying Chen, Xiaohong Xin, Menghan Liu, Yuan Ma, Yifei Ren, Jing Ji, Qi Yu, Lei Qu, Suxia Wang, Gang Liu, Chengang Xiang, Li Yang

**Affiliations:** ^1^ Renal Division, Renal Pathology Center, Peking University First Hospital, Beijing, China; ^2^ Institute of Nephrology, Peking University, Beijing, China; ^3^ Key Laboratory of Renal Disease, Ministry of Health of China, Beijing, China; ^4^ Key Laboratory of CKD Prevention and Treatment, Ministry of Education of China, Peking University, Beijing, China; ^5^ Research Units of Diagnosis and Treatment of Immune-mediated Kidney Diseases, Chinese Academy of Medical Sciences, Beijing, China; ^6^ Laboratory of Electron Microscopy, Pathological Center, Peking University First Hospital, Beijing, China

**Keywords:** human amniotic epithelial cells (hAECs), exosomes, cisplatin, acute kidney injury, chemotherapy

## Abstract

**Background:** Cisplatin is a widely used chemotherapeutic drug, whereas the clinical application is greatly limited by its nephrotoxic side effect. Currently, there has been no effective treatment to prevent cisplatin-induced acute kidney injury (cisplatin-AKI). Human amniotic epithelial cells (hAECs) and their derived exosomes (EXOs) have been proven to effectively protect against ischemia reperfusion-induced AKI, yet their roles in cisplatin-AKI are still unknown.

**Methods:** C57BL/6J mice were given two doses of cisplatin at 20 or 15 mg/kg of body weight to induce AKI with or without mortality. hAECs or EXOs were injected via tail vein 1 day after cisplatin administration. Serum and kidney tissues were collected on the fourth day after 15 mg/kg cisplatin treatment to explore the nephro-protective effects of hAECs and EXOs on cisplatin-AKI. Lung cancer xenograft model was built by subcutaneous injection of A549 cells into BALB/c nude mice to evaluate the effect of hAECs or EXOs on cisplatin chemotherapy.

**Results:** Cisplatin nephrotoxicity was significantly attenuated by hAECs and EXOs as evidenced by reduced mortality rate and decreased serum creatinine (sCr) and reduced tubular injury score. hAECs or EXOs exerted the nephro-protective effects via suppression of TNF-α/MAPK and caspase signaling pathways. In the A549 lung cancer xenograft mouse model, administration of hAECs or EXOs did not promote tumor growth or compromise the therapeutic effects of cisplatin on tumors.

**Conclusion:** This study is the first to demonstrate that hAECs and their derived exosomes have nephro-protective effects in cisplatin-AKI *in vivo*. Importantly, neither hAECs nor EXOs compromise the antitumor activity of cisplatin. These results potentially support the use of hAECs and their derived EXOs as nephro-protectors against cisplatin-induced nephrotoxicity clinically.

## Introduction

Acute kidney injury (AKI) is defined as an abrupt decrease in kidney function within hours, which encompasses structural damage of the kidney and loss of renal function (Makris and Spanou, 2016). It is a common clinical syndrome that complicates the course and worsens the outcome in a significant number of hospitalized patients including patients with tumors (Sanchez and Francoz, 2021). Cis-diamminedichloroplatinum (II) (cisplatin), is one of the most effective chemotherapeutic drugs for a variety of malignant tumors, such as non-small cell lung cancer (NSCLC), head and neck malignancies, bladder cancer, advanced gastric cancer, metastatic triple-negative breast cancer, etc., (Dasari and Tchounwou, 2014). However, the nephrotoxic side effects of cisplatin greatly restrict its clinical application (Volarevic et al., 2019). Cisplatin is mainly taken up and excreted through proximal tubule-localized transporters, such as OCT2 and MATE1 (Ciarimboli, 2014). Consequently, cisplatin accumulates in renal proximal tubular cells, resulting in inflammation, injury, and cell death (Holditch et al., 2019). It is reported that the incidence of AKI in cancer patients after receiving cisplatin chemotherapy reaches about 20%–40% (Arany and Safirstein, 2003; dos Santos et al., 2012), of which about 13% of patients develop AKI after the first course of cisplatin chemotherapy. Therefore, there is an urgent need for the clinical application of safe and efficacious nephro-protective therapy for cisplatin-treated patients (Motwani et al., 2018).

In recent years, stem cell therapy has shown great potential in the treatment of AKI (Barnes et al., 2016; Sávio-Silva et al., 2020). Allogeneic or xenotransplantation of mesenchymal stem cells (MSCs) from different sources (such as bone marrow, fat, cord blood, etc.,) has been reported to have beneficial therapeutic effects on cisplatin induced AKI (cisplatin-AKI) (Sávio-Silva et al., 2020). Despite the supportive results, the use of MSCs in cisplatin-AKI still encounters the risks of tumorigenicity and promotion of tumor cell proliferation. Human amniotic epithelial cells (hAECs) are epithelial cells isolated from the amniotic membrane tissue on the side of the placenta near the fetus. hAECs have the ability to differentiate into cells of all three germ layers, namely, ectoderm, mesoderm, and endoderm (Miki et al., 2005). Studies have shown that hAECs have the advantages of low mutation frequency, low immunogenicity, lack of telomerase leading to non-tumorigenicity, abundant cell sources, no ethical risks, etc., which have attracted widespread attention in regenerative medicine (Miki, 2018).

Recent studies have shown that the application of hAECs in animal models of many diseases has achieved good therapeutic effects, including lung injury and liver fibrosis (Cargnoni et al., 2018; Tan et al., 2018). It has been reported that in chemotherapy-induced premature ovarian failure, exosomes (EXOs) derived from hAECs can inhibit apoptosis by transferring miRNAs and restore ovarian function (Zhang et al., 2019). Combining the limitations of previous stem cell therapy on cisplatin-AKI and the advantages of hAECs, this study aims to explore the therapeutic effects of hAECs and EXOs on cisplatin induced acute nephrotoxicity, and to systematically evaluate the safety of hAECs or EXOs on tumor proliferation and on the effect of cisplatin chemotherapy in NSCLC xenograft mouse model.

## Methods

### Experimental Animals

Male C57BL/6J mice (7 weeks old) and BALB/c nude mice (4 weeks old) were purchased from Vital River Laboratory Animal Technology (Beijing, China) [License No. SCXK (Jing) 2016–0011]. All mice were maintained in animal facilities under specific pathogen-free (SPF) conditions. All animal experiments were performed with the approval of the Institutional Animal Care and Use Committee of Peking University First Hospital (Approval Number: J202065).

### Isolation and Culture of hAECs

hAECs were provided by Shanghai iCELL Biotechnology Co., Ltd. (Shanghai, China). The isolation and culture of hAECs had been described in our previous study (Ren et al., 2020). Briefly, hAECs were isolated from fresh amnion membranes collected from healthy mothers after cesarean deliveries with written and informed consent. The procedure was approved by the Institutional Ethics Committee of the International Peace Maternity and Child Health Hospital, School of Medicine, Shanghai Jiao Tong University (Approval Number: [2014]11). hAECs were cultured by complete culture medium (DMEM/F12 supplemented with 10% FBS, 2 mM glutamine, 1% streptomycin-penicillin, and 10 ng/ml recombinant human epidermal growth factor) in 5% CO2 incubated at 37°C. The P1 hAECs resuspended in PBS at 1 × 10^7^/ml were used in the follow-up experiments.

### Isolation and Identification of EXOs

The isolation and identification of hAECs derived EXOs have been described in our previous study (Ren et al., 2020). Briefly, when cultured hAECs were 80%–90% confluent, the complete culture medium was replaced with serum-free DMEM/F12 medium. After 24 h, the conditioned culture medium was collected and experienced serial centrifugation at 2,000 × *g* for 30 min at 4°C, and 20,000 × *g* (Beckman Coulter, USA) for 30 min at 4°C and 150,000 × *g* for 70 min at 4°C twice to obtain EXOs. The ultrastructure of the EXOs was analyzed under a transmission electron microscope (Zeiss, Oberkochen, Germany). The protein levels of exosome markers CD63, TSG101, Alix, and Flotllin were detected using Western blots. Nanoparticle tracking analysis (NTA) was performed to determine the size and concentration of the purified vesicles (Particle Metrix, Meerbusch, Germany).

### Cisplatin-AKI in C57BL/6J Mice

After adaptive feeding for 1 week, 7-week-old male C57BL/6J mice were randomly divided into four groups: PBS group, cisplatin group, cisplatin + hAECs group, and cisplatin + EXOs group. Each group contained 9 or 10 mice. A single intraperitoneal injection of cisplatin (Cat# P4394, Sigma Aldrich, USA) was used to induce AKI. Cisplatin at 20 mg/kg of body weight was used to induce severe AKI with mortality after 3 days post cisplatin injection (Holditch et al., 2019). Cisplatin at 15 mg/kg of body weight was used to induce moderate AKI without mortality. The normal control mice were administered the same volume of PBS. At 24 h after cisplatin injection, 100 μl of PBS, hAECs (1 × 10^6^), or EXOs (1 × 10^8^) resuspended in PBS was injected into the mice intravenously. The dosages of hAECs and EXOs in the treatment groups were determined according to our previous study (Ren et al., 2020). Blood and kidney samples were collected from moderate cisplatin-AKI mice 4 days after cisplatin injection. Serum creatinine (sCr) levels were measured by the creatinine assay kit (Cat# DICT-500, BioAssay Systems, USA) according to the improved Jaffe method.

### Renal Histology

Kidney tissue sections were fixed with 10% buffered formalin followed by paraffin embedding and stained with periodic acid-Schiff (PAS). The degree of tubulointerstitial damage was scored semi-quantitatively by two renal pathologists who were blinded to the experimental groups. The scores were based on a 0 to 4 + scale, according to the percentage of the cortex and outer medullar region affected by loss of brush border, tubular necrosis, and renal tubular cell cast (0 = no lesion, 1+ = < 25%, 2+ = >25%–50%, 3+ = >50%–75%, 4+ = >75% to <100%).

### NSCLC Tumor Model in Nude Mice

A549, a cell line of non-small cell lung cancer (NSCLC), was purchased from the American Type Culture Collection. A549 was cultured in high-glucose DMEM with 10% FBS and 1% penicillin–streptomycin. To establish NSCLC xenografts, 1 × 10^6^ A549 suspended in 50 μl of PBS added with 50 μl of Matrigel (Cat#356234, BD, USA) was subcutaneously injected at the right flank of male BALB/c nude mice to grow tumor for about 1 month. Measurements of tumor volume were calculated using the following formula: volume (mm^3^) = [the longer diameter × (the shorter diameter)^2^]/2. When the volume of the NSCLC xenografts reached to about 80 mm^3^, tumor-bearing nude mice were randomly assigned to six groups to receive PBS, hAECs, EXOs, cisplatin, cisplatin + hAECs, or cisplatin + EXOs. A single intraperitoneal injection of cisplatin was conducted on nude mice at a dose of 10 mg/kg; 24 h after cisplatin injection, hAECs (1 × 10^6^/100 μl) or EXOs (1 × 10^8^/100 μl) resuspended in PBS were injected into the mice intravenously. Tumor volumes were measured every 4 days *in situ.* Nude mice were sacrificed on day 12 after cisplatin injection to collect NSCLC tumors and mouse kidneys.

### RNA Sequencing

On day 4 after cisplatin treatment, kidney samples of two randomly selected C57BL/6J mice in each group and tumor samples from two randomly selected nude mice with NSCLC in each group, including PBS group, cisplatin group, cisplatin + hAECs group, and cisplatin + EXOs group, were extracted for RNA sequencing. Briefly, total RNA of tissues was extracted, the integrity and purity of RNA were detected by an Agilent 2,100 Bioanalyzer system, and the cDNA libraries constructed by polymerase chain reaction amplification were sequenced by Novaseq, the Illumina High-Throughput Sequencing Platform. Cutadapt (v1.10) was used to filter low-quality reads. When the N content of any sequencing read was more than 10% of the number of bases of the read, these paired reads were removed. When the number of low quality (Qphred≤5) bases in any of the sequencing reads was more than 50% of the number of bases in the read, these paired reads were removed. The clean reads were aligned to the UCSC mm10 (the kidneys of C57BL/J mice) or hg19 genome (the tumors of mice with A549 NSCLC) by the HISAT2 program. The edgeRSeq algorithms were applied to recognize significantly differentially expressed genes with the following criteria: log2 fold-change > 2 or < −2.

Gene Ontology (GO) (http://www.geneontology.org) analysis was conducted to construct the main function of the differentially expressed mRNAs (*p*-value <0.05). The −log 10 (*p*-value) value with the enrichment score represents the significance of the GO term. The Kyoto Encyclopedia of Genes and Genomes (KEGG) pathway analysis (http://www.genome.jp/kegg/) was performed to harvest pathway clusters covering differentially regulated mRNA profiles in the molecular interaction networks. The −log 10 (*p*-value) value with the enrichment score indicates the significance of correlation in the pathway.

### Cell Counting kit-8 Assay

HK-2, the immortalized human proximal tubular cells, were cultured in DMEM with 10% FBS and 1% penicillin–streptomycin under a humidified atmosphere consisting of 5% CO_2_ and 95% air at 37°C. When the degree of cell fusion reached about 70%, cisplatin injury was induced by treating the cells with 10 mM cisplatin for 48 h, and as for the treatment of EXOs, a concentration of 1 × 10^8^/ml EXOs was added to DMEM culture medium after cisplatin treatment.

For cell growth assays, 5 × 10^3^ cells per well were seeded into 96-well plates, with six wells used for each assayed group. After 48 h treatment of cisplatin and/or EXOs, cell numbers were evaluated using the cell counting kit-8 (CCK-8) (Cat# CK04, Dojindo, Japan). Ten microliters of CCK-8 reagent were added to each well, after which the plate was incubated at 37°C for 2 h. Subsequently, the absorbance at 450 nm was measured in each well by using a spectrophotometer (Bio-rad, USA).

### Terminal Deoxynucleotidyl Transferase-Mediated dUTP Nick End-Labeling Assay

Apoptosis in kidney tissues and HK2 cells was detected by the *in situ* terminal deoxynucleotidyl transferase-mediated dUTP nick end-labeling (TUNEL) method following the standard protocol (Cat# C1088, Beyotime, China). Six to ten high-power fields were selected randomly from each slide and the number of TUNEL-positive cells was determined per field by Nikon 90i microscope.

### Immunofluorescence Staining

Immunofluorescence staining of the kidneys was performed on paraffin sections. After fixation and antigen retrieval, nonspecific binding was blocked with 3% BSA. Tissue sections were incubated with the mixed primary antibodies of rabbit anti-Ki67 (1:400, Cat# 9129, CST, USA) and mouse anti-Ecadherin (1:300, Cat# 14472, Abcam, USA) overnight at 4°C. After washed three times with PBS, the tissue sections were incubated with mixed secondary antibodies of Cy3-labeled goat anti-rabbit (1:500, Cat# A0516, Beyotime, China) and FITC-labeled goat anti-mouse (1:500, Cat# A0568, Beyotime, China). After another washing with PBS, the sections were coverslipped with fluorescent mounting medium with DAPI (4,6-diamidino-2-phenylindole) (Cat# ZLI-9557, ZSGB-BIO, China). The staining was examined using fluorescence microscope (Leica, Germany). All images were acquired using the same microscope and camera set. Six to ten high-power fields from the outer medulla and cortex in each kidney examined were captured, then the number of Ki67-positive cells was counted, and the fluorescence intensity of Ecadherin was measured by Image Pro Plus (Media Cybernetics, USA).

### Immunohistochemistry Staining

Immunohistochemistry staining of the kidney was performed on paraffin sections. The primary antibodies included rabbit anti-Ly6G (1:2000, Cat# 238132, Abcam, USA) and rabbit anti-F4/80 (1:200, Cat# 70076, CST, USA). The slides were then exposed to DAB-labeled secondary antibodies. Six to ten fields (×400) were selected randomly from each section, and the staining was examined using a microscope (Leica, Germany).

### Western Blots

Total protein from kidney samples of C57BL/6J mice or HK2 cells was extracted on ice with RIPA buffer (Cat# P0013B, Beyotime, China) supplemented with protease and phosphatase inhibitor cocktail (Cat# P1046, Beyotime, China) following standard protocols. Protein concentration was measured using a Pierce BCA Protein Assay kit (Cat# 23227, Thermo Fisher Scientific, USA). Next, denatured proteins were separated in sodium dodecyl sulfate-polyacrylamide gels and then were electrically transferred onto polyvinylidene difluoride membranes. The membranes were blocked with 5% BSA in Tris-buffer saline with 0.1% Tween 20 (TBST) for 1 h at room temperature and incubated with primary antibodies at 4°C overnight. The following primary antibodies and dilutions were used: anti-kidney injury molecule 1 (KIM-1) (1:1000, Cat# 233720, Abcam, USA), anti-TNF-α (1:1000, Cat# 11948, CST, USA), anti-p-ERK (1:1000, Cat# 4370, CST, USA), anti-ERK (1:1000, Cat# 4695, CST, USA), anti-p-p38 (1:1000, Cat# 4511, CST, USA), anti-p38 (1:1000, Cat# 8690, CST, USA), anti-p-JNK (1:1000, Cat# 4668, CST, USA), anti-JNK (1:1000, Cat# 9252, CST, USA), anti-cleaved caspase 3 (1:500, Cat# 9661, CST, USA), anti-caspase3 (1:1000, Cat# 9662, CST, USA), anti-β-actin (1:1000, Cat# 4970, CST, USA), and anti-GAPDH (1:10000, Cat# 181602, Abcam, USA). After washing three times with TBST, the membranes were incubated with horseradish peroxidase (HRP)-conjugated secondary antibodies for 1 h at room temperature. After three washes with TBST, the membranes were incubated in Immobilon® ECL Ultra Western HRP Substrate (Cat# WBULS0500, Millipore, USA), and images were captured by an ImageQuant LAS 4000 mini system (GE, Healthcare). The relative intensity of the protein bands was quantified by digital densitometry using ImageJ software (Media Cybernetics, USA). The level of GAPDH or β-actin was used as an internal standard.

### RT-PCR

Kidney tissues were collected in RNase-free tubes, and total RNA was isolated using TRIzol® reagent following the instructions of the manufacturer (Cat# 15596026, Thermo Fisher, USA). RNA concentrations were determined by photometric measurements. cDNA was synthesized from 2 μg of total RNA using Fast King RT Enzyme (Cat# KR118, TIANGEN, China) for real-time RT-PCR. The mRNA expression levels of *Ccl2*, *Cxcl1*, *Cxcl2*, *IL-1β*, *IL-6*, and *Gapdh* were determined using SYBR Green PCR Master Mix (Cat# FP209, TIANGEN, China) based on the instructions of the manufacturer with the following primers. All PCR analyses were performed on an ABI Vii7 system. The comparative gene expression was calculated by the 2−ΔΔCt method.

**Table:** 

Gene	Forward primers (5′-3′)	Reverse primers (5′-3′)
*Ccl2*	TTA​AAA​ACC​TGG​ATC​GGA​ACC​AA	GCA​TTA​GCT​TCA​GAT​TTA​CGG​GT
*Cxcl1*	CTG​GGA​TTC​ACC​TCA​AGA​ACA​TC	CAG​GGT​CAA​GGC​AAG​CCT​C
*Cxcl2*	CCA​ACC​ACC​AGG​CTA​CAG​G	GCG​TCA​CAC​TCA​AGC​TCT​G
*Il-1β*	GCA​ACT​GTT​CCT​GAA​CTC​AAC​T	ATC​TTT​TGG​GGT​CCG​TCA​ACT
*Il-6*	TAG​TCC​TTC​CTA​CCC​CAA​TTT​CC	TTG​GTC​CTT​AGC​CAC​TCC​TTC
*Gapdh*	AGG​TCG​GTG​TGA​ACG​GAT​TTG	TGT​AGA​CCA​TGT​AGT​TGA​GGT​CA

### Statistical Analysis

Statistical analysis was performed using SPSS 25.0 statistical software (SPSS, USA). Normally distributed variables are expressed as mean ± SEM and compared using a *t*-test. Non-normally distributed nonparametric variables are expressed as median and interquartile range and are compared between groups using the Mann–Whitney *U*-test. All *p*-values were two-tailed, and *p *< 0.05 was considered statistically significant. GraphPad Prism 8.0 was used to form the vector diagrams (GraphPad, USA).

## Results

### hAECs and Their Derived EXOs Ameliorated Cisplatin-AKI in C57BL/6J Mice

**FIGURE 1:**
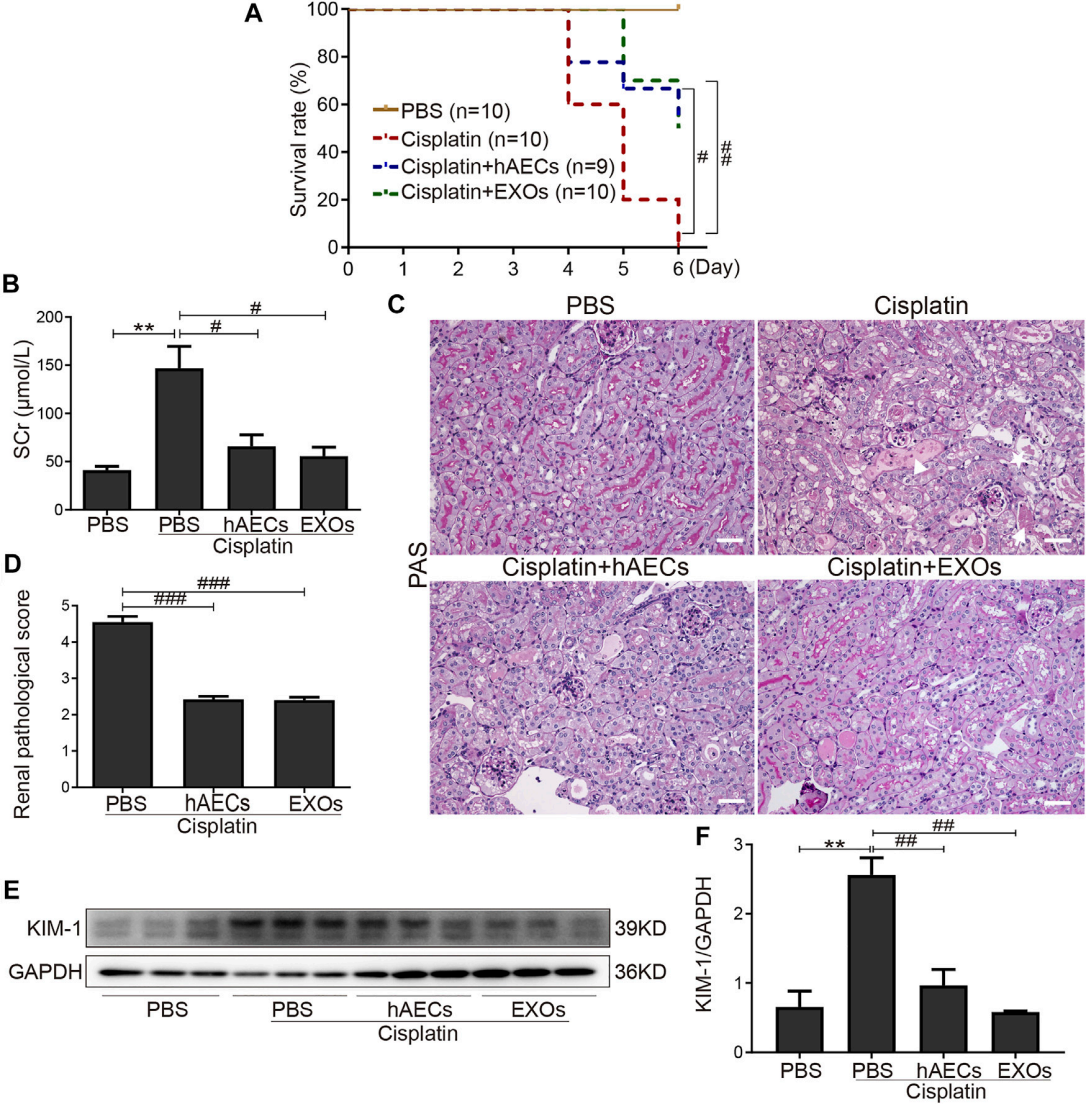
Effects of hAECs or EXOs on C57BL/6J mice with cisplatin-AKI. **(A)** The 6-day mortality rate in mice treated with 20 mg/kg cisplatin (*n* = 9 or 10 mice in each group). **(B)** sCr levels of mice treated with 15 mg/kg cisplatin (*n* = 10). **(C)** Representative micrographs of PAS staining of kidneys from mice with 15 mg/kg cisplatin treatment. Triangle indicates renal tubular necrosis, pentagram indicates the shedding of the brush edge of renal tubules, and arrow indicates the formation of renal casts. Scale bar, 100 μm. **(D**) Semiquantitative renal pathological scores of mice treated with 15 mg/kg cisplatin (*n* = 3). **(E)** Representative Western blots showing protein expression of KIM-1 in kidneys from mice treated with 15 mg/kg cisplatin. **(F)** The relative protein expression of KIM-1 to GAPDH in different groups (*n* = 3). Data are shown as mean ± SEM. **p < 0.01 vs. PBS group, #*p* < 0.05, ##*p* < 0.01, and ###*p *< 0.001 vs. cisplatin group. hAECs, human amniotic epithelial cells; EXOs, exosomes; cisplatin-AKI, cisplatin induced acute kidney injury; sCr, serum creatinine; PAS, periodic acid-Schiff; KIM-1, anti-kidney injury molecule 1.

To establish the roles of hAECs and their derived EXOs in cisplatin acute nephrotoxicity, we examined the mortality, renal function, and renal tissue damage in cisplatin-AKI mice with hAECs or EXOs administration 1 day post cisplatin injection. The 6-day mortality rate in high-dose cisplatin (20 mg/kg)-treated group reached to 100%, while hAECs or EXOs administration significantly decreased the 6-day mortality rate to 50% (Figure 1A). In the 15 mg/kg cisplatin-treated mice, renal function was evaluated on day 4 post cisplatin injection, and a significant high level of sCr was detected (146.7 ± 22.8 μmol/L vs. 41.1 ± 4.0 μmol/L in normal mice, *p* = 0.006). Administration of hAECs or EXOs substantially improved the renal function (*p *< 0.05) with sCr levels of 65.7 ± 12.0 μmol/L and 55.5 ± 9.4 μmol/L, respectively, (*p* < 0.05 vs. cisplatin group) (Figure 1B).

We next examined the renal histology. As shown in Figure 1C, PAS staining revealed severe tubular injury reflected by dilation, necrosis, cast formation, and loss of the brush border in the renal cortical region of mice treated with 15 mg/kg of cisplatin. hAEC- or EXO-treated mice showed significantly improved renal histology with decreased renal pathological scores compared with the cisplatin-AKI mice (2.4 ± 0.1 vs. 2.4 ± 0.1 vs. 4.6 ± 0.2, *p *< 0.001) (Figure 1D). Western blots showed that the protein level of KIM-1 in kidneys of cisplatin-treated mice were markedly reduced when treated with hAECs and EXOs (Figures 1E, F). All these data suggested that hAECs and EXOs could attenuate cisplatin-induced renal dysfunction and pathological damage, exhibiting the renal protective effects on cisplatin-AKI.

### Molecular Modifications by hAECs and Their Derived EXOs in Cisplatin-injured Kidneys

**FIGURE 2:**
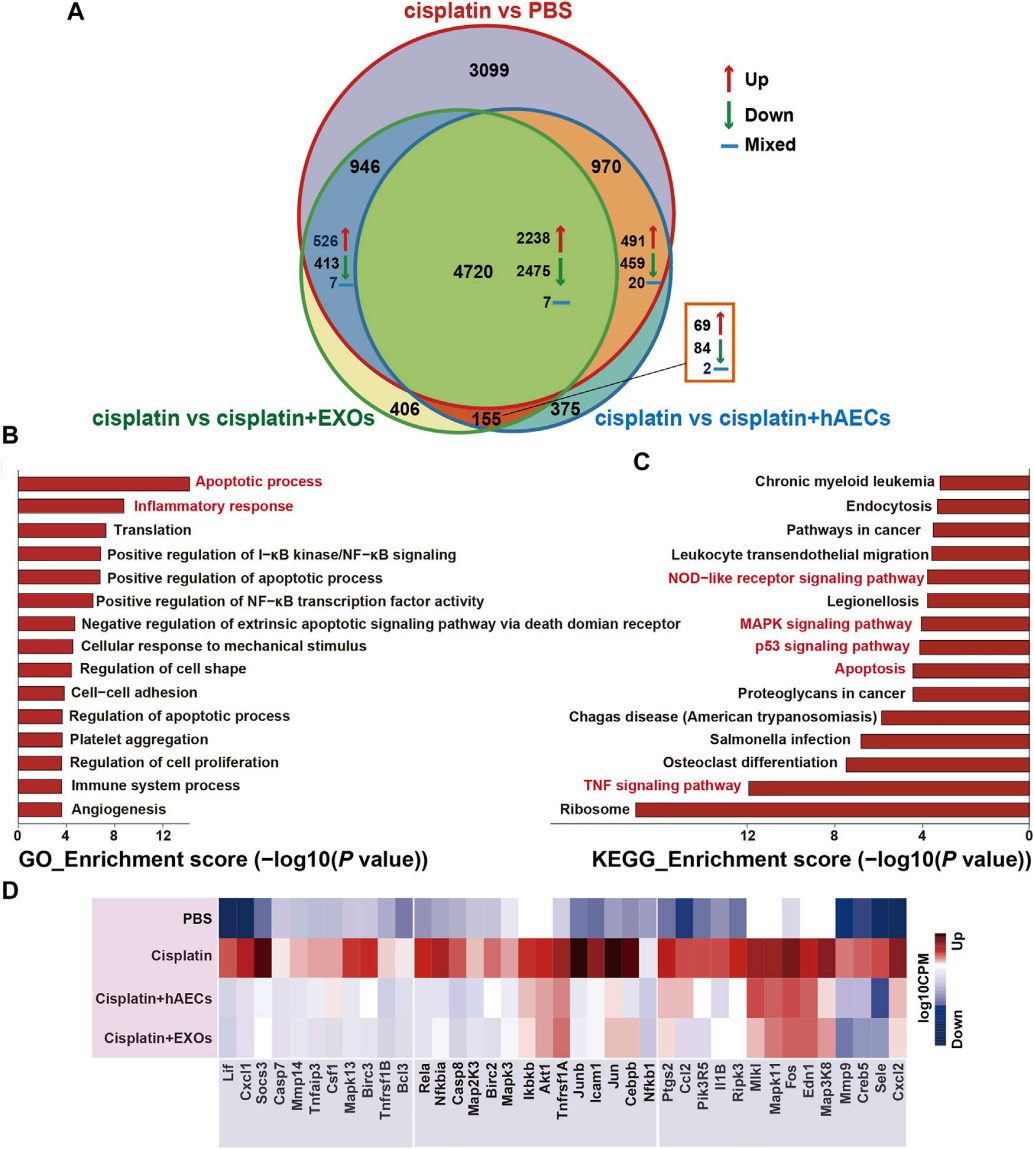
RNA sequencing analysis of kidneys from C57BL/6J mice treated with 15 mg/kg cisplatin. **(A)** Venn diagram showing the numbers of significant DEGs across the four indicated groups. **(B,C)** The top 15 enriched GO terms and KEGG pathways of the 2,238 DEGs. **(D)** Heatmap showing the TNF pathway-related DEGs differentially expressed across the four groups. The colors indicate the value of log10 CPM. CPM, counts per million; cisplatin, Cis-diamminedichloroplatinum (II); DEGs, differentially expressed genes; GO, Gene Ontology; KEGG, Kyoto Encyclopedia of Genes and Genomes.

To explore the mechanisms of hAECs and EXOs in protecting against cisplatin-AKI, we conducted a genome-wide transcriptomic sequencing of normal kidneys, cisplatin-injured kidneys, and cisplatin-injured kidneys treated with hAECs or EXOs. The log10 counts per million (CPM) value was used to quantify the mRNA expression. We analyzed the profiles of differentially expressed genes (DEGs) among the four groups. As shown in Figure 2A, compared with normal kidneys (PBS), totally 9,735 DEGs were found in cisplatin-AKI kidneys (cisplatin). Compared with the cisplatin group, a total of 6,220 DEGs were found in cisplatin-injured kidneys treated with hAECs (cisplatin + hAECs) and 6,227 DEGs in cisplatin-injured kidneys treated with EXOs (cisplatin + EXOs). Four thousand seven hundred twenty genes were found to be significantly changed among the four groups, including 2,238 DEGs upregulated in cisplatin vs. PBS comparison while concomitantly downregulated in the comparison between cisplatin vs. cisplatin + hAECs or cisplatin + EXOs. These data indicated that the molecular regulation of hAECs and EXOs on cisplatin renal injury was very similar, and hAECs and EXOs could counteract on about half of the DEGs regulated by cisplatin injury.

The NIH Database for Annotation, Visualization, and Integrated Discovery (DAVID) was used to perform gene functional annotation clustering on the 2,238 identified DEGs. We focus on the GO terms of biological process since it is the most widely used subontology of GO to evaluate the functions of genes. Accordingly, we obtained 83 significant GO terms in the 2,238 DEGs. The top 15 GO terms of upregulated genes with an adjusted *p*-value <0.05 are shown in Figure 2B. Apoptotic process and inflammatory response were the most enriched GO terms of biological processes in these DEGs. The 2,238 DEGs were also analyzed by KEGG pathway analysis, and the top 15 significant KEGG pathways are shown in Figure 2C, including TNF signaling pathway, apoptosis, p53 signaling pathway, MAPK signaling pathway, and NOD-like receptor signaling pathway. TNF signaling pathway was reported to play a central role in cisplatin-induced renal inflammation (Holditch et al., 2019). We next explored TNF pathway-related DEGs with different expression directions between the cisplatin group and the cisplatin + hAECs or cisplatin + EXOs groups. As shown in Figure 2D, the TNF-associated DEGs including MAPK cascade compartments *Tnfaip3*, *Mapk3*, *Mapk13*, *Jun*, and the downstream effectors such as *Cxcl1*, *Cxcl2*, *Ccl2*, *Csf1*, *Socs3*, *Mmp9*, *Mmp14*, *Il1b* were upregulated in the cisplatin group compared with PBS control, but those genes were all downregulated in cisplatin + hAEC and cisplatin + EXO groups compared with the cisplatin group.

### hAECs and Their Derived EXOs Alleviated Cisplatin-Induced Kidney Inflammation by Inhibiting TNF-α/MAPK Signaling Pathway

**FIGURE 3:**
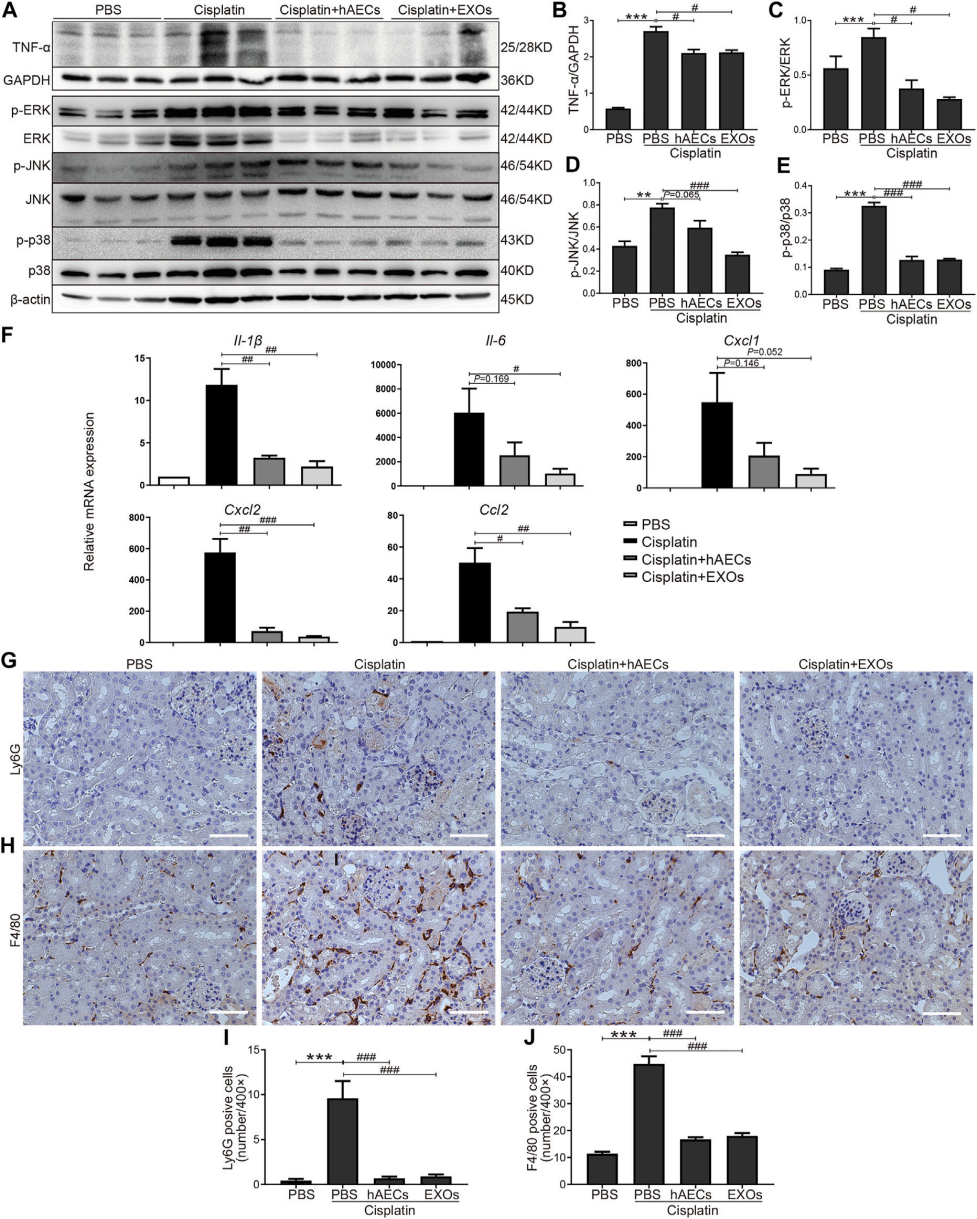
Regulation of TNF-α/MAPK pathway by hAECs and EXOs in C57BL/6J mice treated with 15 mg/kg cisplatin. **(A)** Representative Western blots showing protein expression of TNF-α, p-ERK, ERK, p-JNK, JNK, p-p38, and p38 of kidneys in different groups as indicated. **(B–E)** The relative protein expression of TNF-α to GAPDH, p-ERK to ERK, p-JNK to JNK, and p-p38 to p38 in different groups (*n* = 3). **(F)** The relative mRNA expression of *Il-1β*, *Il-6*, *Cxcl1*, *Cxcl2*, and *Ccl2* determined by RT-PCR. **(G,I)** Representative micrographs and quantification of Ly6G positive staining on kidney sections of different groups as indicated. **(H,J)** Representative micrographs and quantification of F4/80 positive staining on kidney sections of different groups as indicated. Scale bar, 100 μm *n* = 3 for each group. Data are shown as mean ± SEM. **p < 0.01 and ***p < 0.001 vs. PBS group, #*p *< 0.05, ##*p *< 0.01, and ###*p *< 0.001 vs. cisplatin group.

Multiple studies have shown that TNF-α-triggered MAPK cascades mediate various cellular responses to cisplatin-induced kidney injury (Jo et al., 2005; Ramesh and Reeves, 2005; Francescato et al., 2007). To further validate the results of RNA sequencing, Western blot was performed to detect TNF-α expression in the homogenates of normal kidneys, cisplatin-treated kidneys, and cisplatin-injured kidneys treated with hAECs or EXOs (Figure 3A). Compared with the PBS control, cisplatin treatment significantly increased the level of kidney TNF-α expression, which was greatly suppressed by hAECs or EXOs administration (Figure 3B). As shown in Figure 3A, the levels of phosphorylated ERK1/2 (*p *< 0.001), JNK (*p *< 0.01), and p38 (*p *< 0.001) were significantly increased in the cisplatin group compared with the PBS control group. As expected, the expression of these proteins was significantly reduced in hAEC- or EXO-treated groups (Figures 3C–E). These data indicated that hAECs or EXOs significantly prevented the activation of MAPK signaling cascade induced by cisplatin. Downstream expression of cytokines/chemokines was also detected. RT-PCR results showed that the expression of inflammatory factors including *Cxcl1*, *Cxcl2*, *Ccl2*, *Il-1β*, and *Il-6* was greatly induced by cisplatin, which was significantly decreased after hAECs or EXOs treatment (Figure 3F). Consequently, the infiltration of immune cells such as neutrophils (Ly6G) and macrophages (F4/80) were decreased in the cisplatin + hAECs and cisplatin + EXO-treated groups compared with the cisplatin group (Figures 3G–J).

### hAECs and Their Derived EXOs Attenuated Cisplatin-Induced Apoptosis in Renal Tubular Epithelial Cells

**FIGURE 4:**
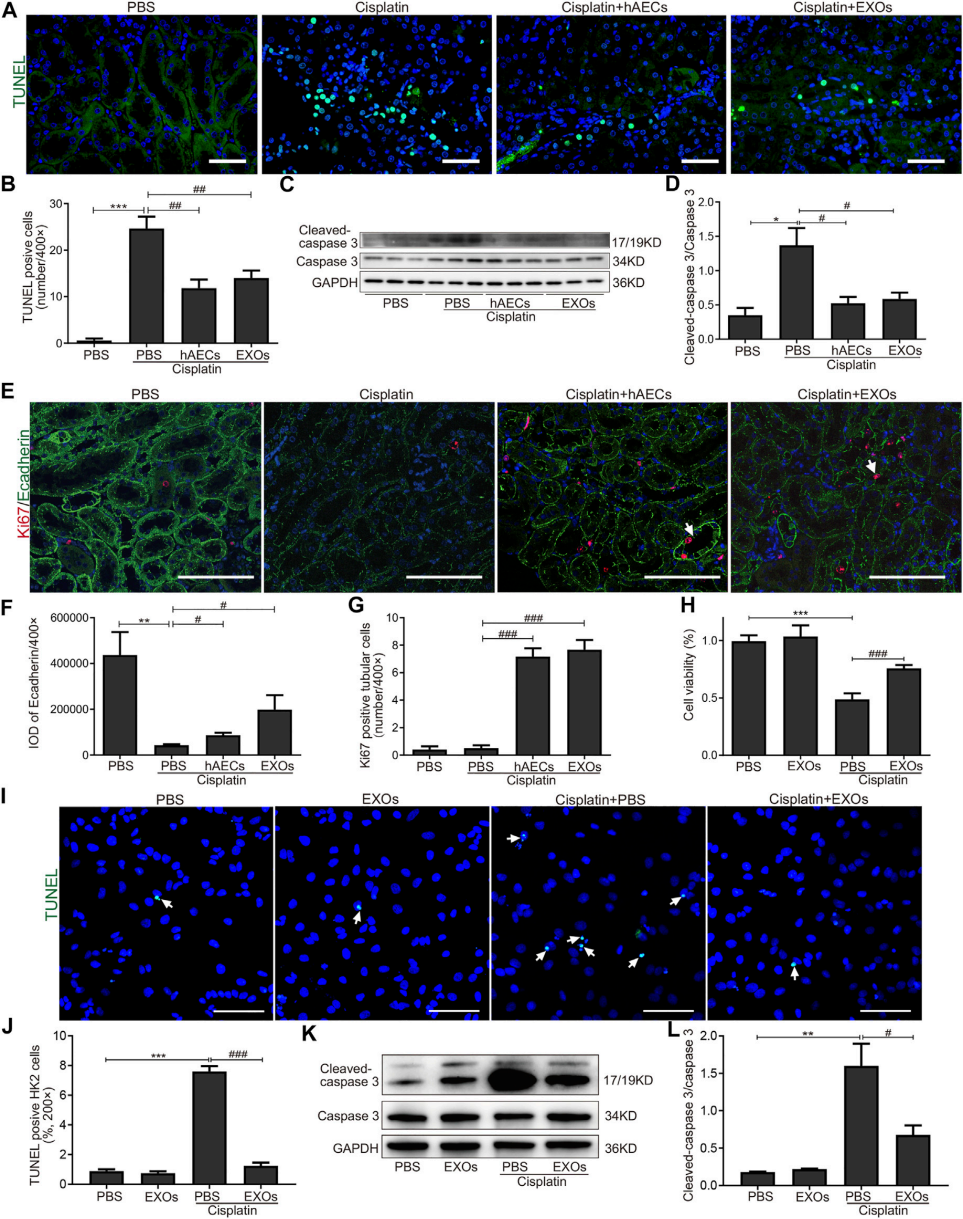
Inhibition of cisplatin-induced apoptosis of renal tubular epithelial cells by hAECs and EXOs. **(A)** Representative micrographs of TUNEL (green) staining of kidneys from C57BL/6J mice treated with 15 mg/kg cisplatin. **(B)** Quantification of TUNEL-positive cells/400×. **(C)** Representative Western blots showing protein expressions of cleaved-caspase 3 and caspase 3 of kidneys from C57BL/6J mice treated with 15 mg/kg cisplatin. **(D)** The relative protein expression of cleaved-caspase 3 to caspase 3 in different groups. *n* = 3 for each group. **(E)** Representative micrographs of Ki67 (red) and Ecadherin (green) immunofluorescence staining of kidneys from C57BL/6J mice treated with 15 mg/kg cisplatin. **(F)** Semiquantitative analysis of intensity of Ecadherin immunofluorescence staining. IOD, integrated optical density. **(G)** Quantification of Ki67-positive tubular cells/400×. **(H)** CCK-8 assay showing the cell viability of HK2 cells with different treatments for 48 h. **(I)** Representative micrographs of TUNEL (green) staining of HK2 cells with different treatments for 24 h. **(J)** Percent of TUNEL-positive cells to all HK2 cells/200×. **(K)** Representative Western blots showing protein expressions of cleaved-caspase 3 and caspase 3 in HK2 cells with different treatments for 48 h. **(L)** The relative protein expression of cleaved-caspase 3 to caspase 3 in HK2 cells with different treatments for 48 h. Scale Bar, 100 μm *n* = 3 for each group. Data are shown as mean ± SEM. *p < 0.05, **p < 0.01, and ***p < 0.001 vs. PBS group, #*p *< 0.05, ##*p *< 0.01, and ###*p *< 0.001 vs. cisplatin group. TUNEL, terminal deoxynucleotidyl transferase mediated dUTP nick end-labeling; CCK-8, cell counting kit-8.

We next evaluated the effects of hAECs and EXOs on the apoptosis and proliferation of kidney cells in cisplatin-AKI. TUNEL assay was performed in mouse kidney sections. As shown in Figure 4A, on the fourth day of cisplatin injection, a large number of TUNEL-positive cells was observed in the cisplatin group, and the number of TUNEL-positive cells were significantly reduced in hAEC- or EXO-treated groups (Figure 4B). Western blot analysis showed that the expression level of apoptosis-related marker cleaved caspase-3 in the cisplatin-treated group was significantly higher than that in the PBS control group, while hAEC and EXO treatment significantly suppressed cleaved caspase-3 expression in the kidneys (Figures 4C, D). We next examined the expression of Ecadherin, a marker for epithelial cell tight junctions, and Ki67 to evaluate the cell viability and proliferation states. As shown in Figure 4E, Ecadherin expression in renal tubular epithelial cells in the cisplatin group was significantly decreased compared with the PBS control group. However, the expression of Ecadherin and Ki67 in hAECs and EXOs treated groups increased significantly, and most of the Ki67 positive cells were located in the renal tubules, suggesting that hAECs and EXOs promoted the renal tubule repair and the proliferation of tubular epithelial cells post-cisplatin injury (Figures 4F, G).

We further cultured HK2 cells *in vitro* to observe whether EXOs could reduce the damage of cisplatin to renal tubular cells. We first assessed cell viability by the CCK8 assay. As shown in Figure 4H, after cisplatin stimulation for 48 h, the survival rate of cells in the EXOs group was significantly higher than that in the cisplatin group. The number of TUNEL positive cells was increased and the cleaved caspase-3 expression was upregulated in HK2 cells for cisplatin treated group. However, the apoptotic cell number and the enhanced protein levels of cleaved caspase-3 in cisplatin-treated HK2 cells were markedly decreased by EXOs treatment (Figures 4I–L). These findings suggested that hAEC and EXO treatment could prevent cisplatin-induced apoptosis and promote proliferation of renal tubular epithelial cells.

### Effects of hAECs and Their Derived EXOs on the Antitumor Efficacy of Cisplatin in a Mouse NSCLC Xenograft Model

**FIGURE 5:**
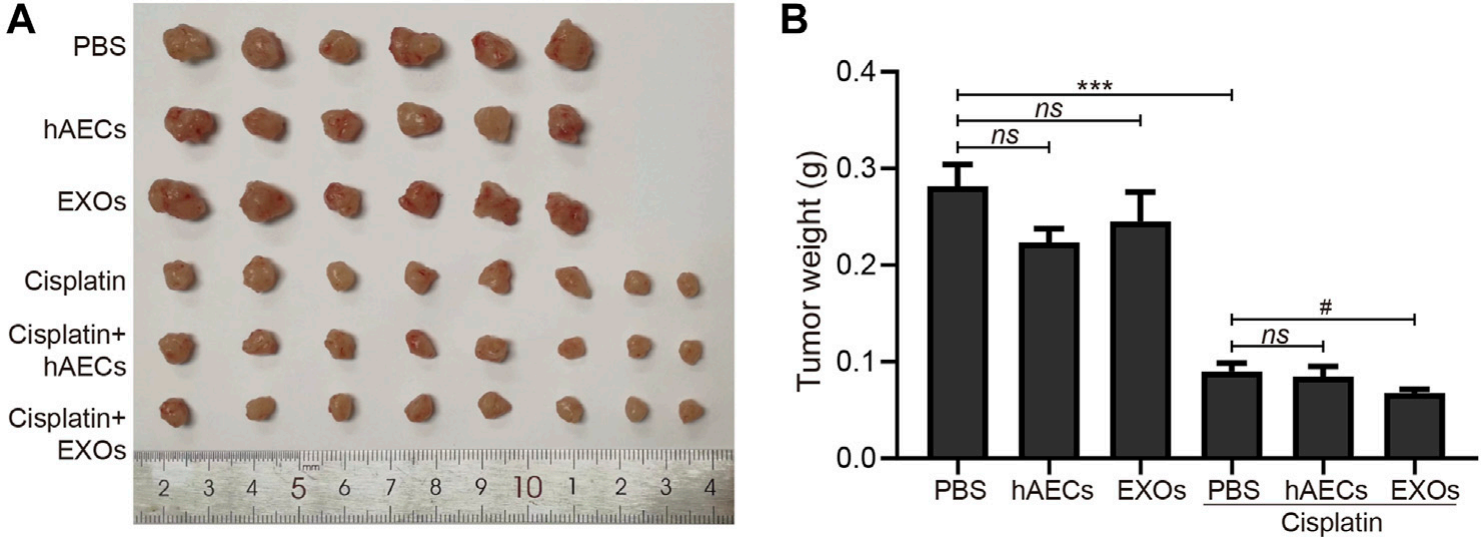
Effects of hAECs and EXOs on the tumor growth in nude mice with A549 NSCLC. **(A)** The gross tumor images in different groups of nude mice with A549 NSCLC on day 12 after cisplatin injection. **(B)** Comparison of tumor weights in different groups of nude mice with A549 NSCLC on day 12 after cisplatin injection. Data are shown as mean ± SEM. ***p < 0.05 vs. PBS group, #*p *< 0.05 vs. cisplatin group. NSCLC, non-small cell lung cancer.

To clarify the effects of hAECs and EXOs on tumor proliferation and anti-tumor effects of cisplatin, we constructed an A549 lung cancer xenograft mouse model. On the 12th day of cisplatin injection, the subcutaneous tumors of A549 in nude mice were collected, photographed, and weighed. As shown in Figures 5A, B, the tumor weight in the hAEC- and EXO-treated groups was similar to that in the PBS group, suggesting that hAECs and EXOs had no potential on promoting A549 lung tumor growth. Meanwhile, the tumor weight in the cisplatin group was significantly lower than that in the PBS group, indicating the treatment efficacy of cisplatin on tumor growth (0.090 ± 0.009 g vs. 0.282 ± 0.023 g, *p < *0.001). Compared with the cisplatin group, the tumor weight in cisplatin + hAECs group (0.085 ± 0.010 g) was similar and in cisplatin + EXOs group was slightly lower (0.068 ± 0.004 g vs. cisplatin group: 0.090 ± 0.009 g, *p < *0.05). These results suggested that hAECs and EXOs did not interfere with the tumor-suppressive effect of cisplatin.

### RNA Sequencing Identified Signaling Pathways Specifically Regulated by hAECs and Their Derived EXOs in Cisplatin-Treated Lung Tumors

**FIGURE 6:**
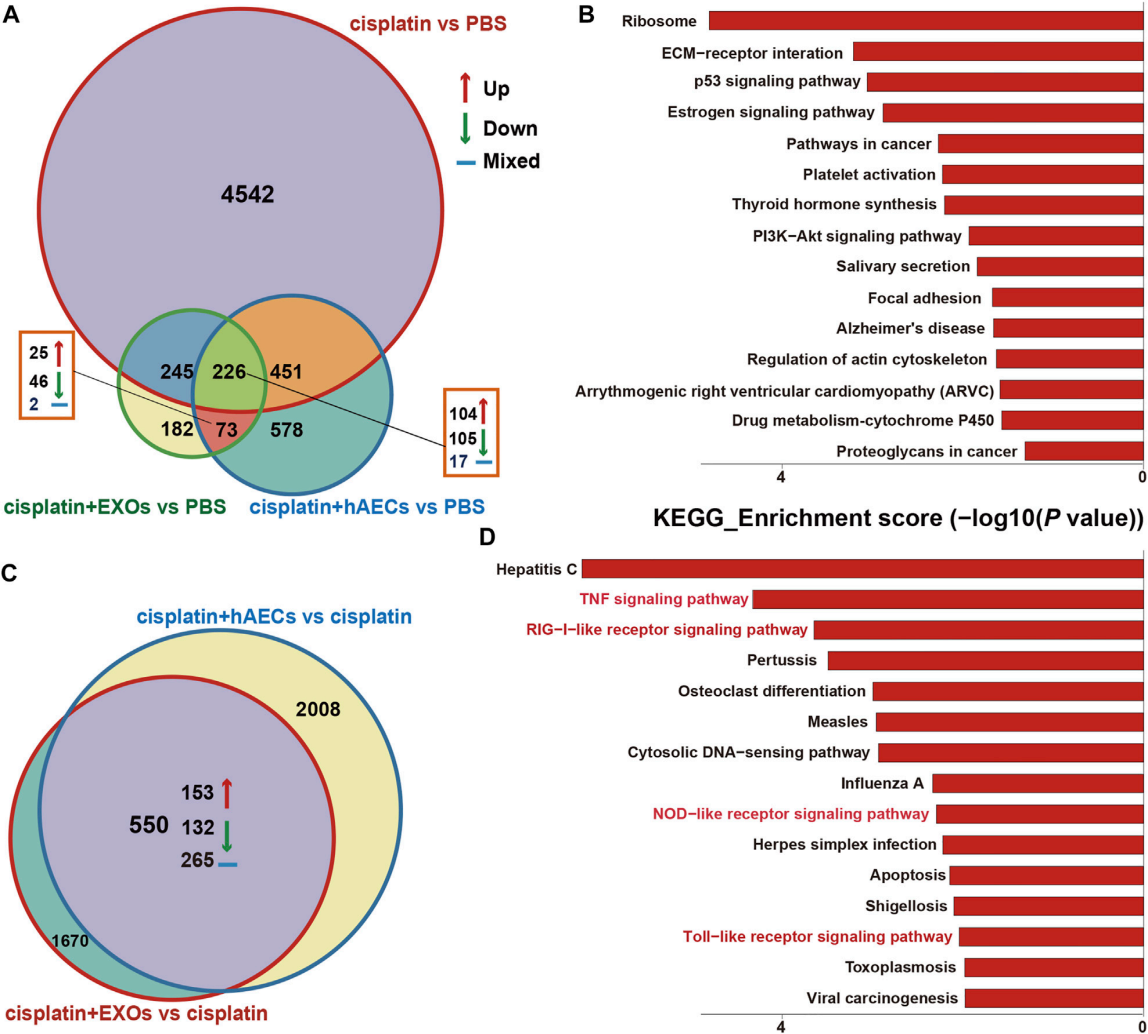
RNA sequencing analysis on tumors from A549 NSCLC nude mice collected 4 days after cisplatin injection. **(A)** Venn diagram showing the numbers of significant DEGs across the four groups as indicated. **(B)** The top 15 enriched and upregulated KEGG pathways in the 226 DEGs across the four groups as indicated in **(A)**. **(C)** Venn diagram showing the numbers of significant DEGs across the two groups as indicated. **(D)** The top 15 enriched and upregulated KEGG pathways in the 550 DEGs across the four groups as indicated in **(C)**.

The RNA of PBS, cisplatin, cisplatin + hAECs, cisplatin + EXO-treated tumors on the fourth day after cisplatin injection was extracted and subjected to RNA sequencing for identification of differentially expressed genes and remarkably changed pathways. When using PBS treatment as the control (Figure 6A), 5,375 DEGs were considered as significantly changed for cisplatin vs. PBS, cisplatin + hAECs vs. PBS, and cisplatin + EXOs vs. PBS. The cisplatin, cisplatin + hAECs, and cisplatin + EXOs groups shared 226 DEGs. The functional associations of the 226 DEGs were implemented by the KEGG analysis (Figure 6B). The ECM–receptor interaction, p53 signaling pathway, estrogen signaling pathway, PI3K–AKT signaling pathway, regulation of actin cytoskeleton, and drug metabolism–cytochrome P450 were the most enriched pathways in the upregulated genes in the 226 DEGs, indicating a common regulation of tumor microenvironment, cell proliferation, cell division, and drug metabolism by cisplatin and hAECs or EXOs. However, when using cisplatin treatment as the control (Figure 6C), 4,228 DEGs were found significantly changed for cisplatin + hAECs vs. cisplatin and cisplatin + EXOs vs. cisplatin. Five hundred fifty DEGs were shared in cisplatin + hAECs and cisplatin + EXOs groups. KEGG analysis showed that TNF signaling pathway, RIG-I-like receptor signaling pathway, NOD-like receptor signaling pathway, and Toll-like receptor signaling pathway were the most enriched pathways in the upregulated 153 genes in the 550 DEGs (Figure 6D). We checked the functions of the 153 upregulated genes and found a serial of genes involved in cell cycle checkpoint control and DNA damage repair, including *Cbr1*, *Ccnb1*, *Cdc6*, *Cdk1*, *Ddias*, *Fignl*, *Mcm3*, *Nek6*, *Nupr1*, and *Psrc1* (Table 1).

**TABLE 1:** Representative DEGs identified by RNA sequencing in cisplatin + hAECs or cisplatin + EXOs compared with cisplatin-treated tumors.

Gene	Cisplatin + hAECs vs. cisplatin		Cisplatin + EXOs vs. cisplatin	
	Log2 FC	*P*_adj_	Log2 FC	*P*_adj_
Adam8	2.9686	3.31 × 10^–9^	0.8359	3.31 × 10^–9^
Bax	1.5060	3.78 × 10^–16^	0.5605	3.78 × 10^–18^
Bcl3	3.0509	1.27 × 10^–32^	2.0370	1.27 × 10^–32^
Brca1	1.9357	5.31 × 10^–5^	1.7134	5.31 × 10^–5^
Bzw2	0.5356	0.045483	0.5295	0.045483
Cbr1	1.9998	1.67 × 10^–44^	0.5610	1.67 × 10^–44^
Ccnb1	3.1586	0.017644	0.8020	0.017644
Cdc6	1.9437	0.021621	1.6689	0.021621
Cdca3	1.3727	0.015941	1.0782	0.015941
Cdca7	1.8503	0.014304	0.6242	0.014304
Cdk1	1.7407	0.000477	0.8007	0.000477
Cldn4	2.7803	9.10 × 10^–38^	1.0485	9.14 × 10^–38^
Clspn	2.5574	0.000734	1.7433	0.000734
Ddias	3.6595	3.69 × 10^–22^	1.1992	3.69 × 10^–22^
Dlgap5	2.5165	0.007954	0.9324	0.007954
Esco2	2.5888	0.000955	1.9327	0.000955
Fignl1	2.3678	0.001049	0.8020	0.001049
Gins2	1.5373	0.001835	1.3674	0.001835
Havcr1	9.3847	1.18 × 10^–91^	1.8328	1.18 × 10^–91^
Irf7	4.0488	4.41 × 10^–162^	0.9286	4.41 × 10^–162^
Lif	5.1139	3.86 × 10^–24^	0.6457	3.86 × 10^–24^
Mcm3	1.0524	0.001352	2.3359	0.001352
Nek6	1.5931	1.73 × 10^–13^	0.7183	1.73 × 10^–13^
Nupr1	1.0746	0.000120	0.9035	0.000120
Psrc1	5.6203	1.56 × 10^–8^	1.6411	1.56 × 10^–8^
Zbp1	2.6423	4.04 × 10^–10^	2.5706	4.04 × 10^–10^
Zmat3	1.4787	4.50 × 10^–19^	1.5122	4.50 × 10^–19^

Note. DEGs, differentially expressed genes; cisplatin, Cis-diamminedichloroplatinum (II); hAECs, human amniotic epithelial cells; EXOs, exosomes.

## Discussion

In recent years, with the increase in the incidence of tumors, the progress of anti-tumor treatments, and the prolonged survival of cancer patients, chemotherapy-related AKI has become more and more common (Sahni et al., 2009; Troxell et al., 2016). Cisplatin, being an effective chemotherapeutic drug, is still widely used in a variety of solid tumors as the first-line chemotherapy. However, cisplatin induced nephrotoxicity greatly limits its clinical application and chemotherapeutic efficacy. According to European guidelines, the only recommended method of preventing cisplatin-AKI in clinic is to intravenously infuse isotonic saline for hydration and supply with magnesium if necessary (Launay-Vacher et al., 2008). It is thus required to explore new methods for the treatment of cisplatin-AKI without interfering with its anti-cancer effects. The reparative therapeutic potential of hAECs have been assessed in a multitude of experimental animal models including lung injury, brain injury, hepatic fibrosis, and multiple sclerosis (Kakishita et al., 2000; McDonald et al., 2015; Cargnoni et al., 2018; Tan et al., 2018). To date, only a few applications of hAECs in renal injury have been reported. Previously our lab has proved that hAECs protected against ischemia–reperfusion AKI in mouse model (Ren et al., 2020). In the current study, we showed that hAECs or their derived EXOs administration in cisplatin-AKI mouse model could reduce mortality, improve renal function, and reduce renal tissue damage. Decreased inflammation and renal cell apoptosis, and increased renal tubular cell proliferation were also observed after hAECs or EXOs administration. Importantly, hAECs treatment did not compromise the antitumor activity of cisplatin in A549 tumor bearing nude mouse model.

More and more evidences indicate that renal tubular epithelial cell apoptosis and renal inflammation mainly determine the progression and outcome of cisplatin-AKI (Volarevic et al., 2019). TNF-α is a pleiotropic pro-inflammatory cytokine inducing a broad range of cellular responses, ranging from inflammatory cytokine production, cell survival, cell proliferation, cell differentiation, and cell death (Chen and Goeddel, 2002). We performed RNA sequencing detection on the kidney tissue of C57BL/6J cisplatin-AKI mice, and found that after cisplatin injury and hAECs or EXOs treatment, the TNF pathway was enriched and upregulated in cisplatin-AKI group but downregulated in cisplatin-AKI mice treated with hAECs or EXOs. TNF-α expression was suppressed after hAECs or EXOs treatment in cisplatin-AKI as shown by Western blot analysis. Once TNF-α binds to the two cell surface receptors, TNFR1 and TNFR2, it can initiate multiple downstream signaling pathways, including NF-κB pathway, MAPK pathway, and exogenous apoptosis pathway (Chen and Goeddel, 2002). The MAPK family mainly includes three phosphorylated proteins, ERK, p38, and JNK (Yue and López, 2020). Our experimental results showed that the phosphorylation of ERK, p38, and JNK was decreased by hAECs or EXOs treatment in cisplatin-AKI, and the expression of apoptosis marker cleaved caspase-3 were also decreased in cisplatin injured HK2 cells treated with EXOs. Together, our data suggest that hAECs and EXOs may inhibit TNF-α production, downregulate the phosphorylation of MAPK signaling molecules, and ultimately inhibit TNF-α-induced inflammatory response and renal tubular cell apoptosis, thereby alleviating cisplatin-AKI.

Increasing studies have suggested that the protective effects of many stem cells, including hAECs, might not be achieved by homing to the injury site and differentiation (Wang et al., 2015; Grange et al., 2019; Lee et al., 2020). For example, in the previous IRI-AKI mouse model, we found that most of the hAECs were trapped in the lungs after tail vein injection, but not in the injured kidneys (Ren et al., 2020). Growing evidences have favored that the stem cell-secreted EXOs are potential carriers of DNA, microRNA, lipids, cell-surface proteins, cytosolic proteins, nucleic acids, amino acids, and metabolites to perform biological functions (He et al., 2018; Kalluri and LeBleu, 2020). In this study, we compared the therapeutic effects of hAECs derived EXOs and hAECs on cisplatin-AKI, and the results showed that EXOs have the same renal protective effects as hAECs. In addition, we found through cultured renal tubular epithelial cells *in vitro* that EXOs could significantly inhibit cisplatin-induced apoptosis and increase the survival rate of tubule cells after cisplatin injury. Our previous work has detected the abundance of extracellular matrix proteins and proteins involved in the IGF signaling, HIF signaling, integrin signaling, Wnt signaling, and TGFβ signaling in hAEC derived exosomes (Ren et al., 2020). EXOs are easy to extract, store, and transport, and have better biocompatibility (Gurunathan et al., 2019). Practically, the use of EXOs, that is, “cell-free” stem cell therapy, may have more advantages than direct infusion of its source stem cells for clinical transformation of cisplatin-AKI stem cell therapy.

The ideal cisplatin-AKI therapy is to protect the kidneys while not compromising the anti-tumor effect of cisplatin and the major concern of implementing MSCs therapy in cisplatin-AKI is their risks of tumorigenicity and the promotion of tumor cell proliferation (Belmar-Lopez et al., 2013; Večerić-Haler et al., 2017; Plava et al., 2020). In the current study, we used the A549 lung cancer cell xenograft mouse model to verify the safety of hAECs or EXOs on tumor growth. The data showed that the tumor weight after hAECs or EXOs administration alone were not significantly different from those in the PBS control group. The combined application of hAECs and cisplatin resulted in no significant change in tumor weight compared with tumors treated with cisplatin alone, while EXOs and cisplatin combination slightly decreased the tumor weight than cisplatin treatment alone. Previously, Kang et al. reported that in a breast cancer MDA-MB-231 cell xenograft mouse model, the application of hAECs resulted in a reduction in tumor volume (Kang et al., 2012). In another BALB/c nude mouse xenograft model, co-injection of hAECs and ovarian cancer cell SK-OV-3 has shown the inhibitory effect on tumor growth (Bu et al., 2017). There are also studies showing that hAECs are non-tumorigenic in immunodeficient mice and healthy volunteers (Akle et al., 1985; Yang et al., 2018). The results from the current study and the previous studies indicate that hAECs and EXOs themselves have no interference with tumor proliferation, and their combined use with cisplatin did not compromise the anti-tumor efficacy of cisplatin.

We then performed RNA sequencing analysis on the tumors to detect the transcriptome difference in the tumors treated with cisplatin combined with hAECs or EXOs with those treated with cisplatin alone. We found that TNF signaling pathway was enriched and upregulated. Further analysis of the RNA-seq data showed that genes closely related to the DNA damage repair (*Bax*, *Bcl3*, *Brac1*, *Fignl1*), DNA replication (*Gins3*, *Mcm3*), and cell cycle progression (*Ccnb1*, *Cdk1*, *Cdc6*, and *Nek6*) were significantly changed with additional hAECs or EXOs treatment (Table 1). TNF-α has been reported to regulate cell cycle progression in different types of cancer cells (Pusztai et al., 1993; Zhang et al., 2018; Jacobson et al., 2019). Recently, studies have shown that combination with low-dose TNF-α could enhance therapeutic effects of chemotherapeutic drugs through the TNF-α/NFκB signaling cascade, driving quiescent cancer cells out of G0/G1 phase to enter treatment sensitive proliferating phases to be killed by chemotherapeutic drugs (Moon et al., 2010; Jayasooriya et al., 2013; Wu et al., 2017). The differential regulation of TNF signaling pathway by hAECs and EXOs in cisplatin injured kidney and in cisplatin treated tumors indicate the complexity of stem cell therapy. The mechanisms of these fine tunings require further investigations.

In the current study, we only tested the therapeutic effects of hAECs or EXOs by single dose injection 1 day after cisplatin administration. Although the results are promising, many challenges remain when translation to clinical application. Based on the significant progress in different types of stem cell therapy (Bagno et al., 2018; Tompkins et al., 2018; Zhang and Lai, 2020), we may achieve improvements on two aspects: one is to identify the beneficial factors carried by hAEC-derived exosomes and to precondition the hAECs to boost their release of therapeutic exosomes; another is to optimize the route of administration, the proper dose and the timing for treatment in order to achieve the goal of complete renal recovery after cisplatin chemotherapy.

## Conclusion

Cisplatin nephrotoxicity is one of the major causes of chemotherapy-related AKI and severe cisplatin-AKI significantly increases the risk of hospital death in patients. Here, we demonstrate that hAECs and their derived EXOs could reduce the mortality rate and attenuate renal dysfunction and pathological damage in the cisplatin-AKI mouse model. The renal protective effects were exerted via inhibition of the TNF-α/MAPK and the caspase signaling pathways without compromising the antitumor activity of cisplatin. Our study may provide new insights for clinical treatment of cisplatin-AKI by stem cell therapy.

## Data Availability

Data of RNA sequencing for all samples has been deposited in Sequence Read Archive (SRA) under accession code PRJNA751451. Other data that support the findings of this study are available from the corresponding author upon reasonable request.
